# Contemporary Review of Smartphone Apps for Tinnitus Management and Treatment

**DOI:** 10.3390/brainsci10110867

**Published:** 2020-11-17

**Authors:** Muntazir Mehdi, Albi Dode, Rüdiger Pryss, Winfried Schlee, Manfred Reichert, Franz J. Hauck

**Affiliations:** 1 Institute of Distributed Systems, Ulm University, 89081 Ulm, Germany; 2 Institute of Databases and Information Systems, Ulm University, 89081 Ulm, Germany; albi.dode@uni-ulm.de (A.D.); manfred.reichert@uni-ulm.de (M.R.); 3 Institute of Clinical Epidemiology and Biometry, University of Würzburg, 97070 Würzburg, Germany; ruediger.pryss@uni-wuerzburg.de; 4 Clinic and Polyclinic for Psychiatry and Psychotherapy, 93053 Regensburg, Germany; winfried.schlee@tinnitusresearch.org

**Keywords:** mobile health, healthcare, mobile apps, tinnitus therapy, cbt, self help, tinnitus research

## Abstract

Tinnitus is a complex and heterogeneous psycho-physiological disorder responsible for causing a phantom ringing or buzzing sound albeit the absence of an external sound source. It has a direct influence on affecting the quality of life of its sufferers. Despite being around for a while, there has not been a cure for tinnitus, and the usual course of action for its treatment involves use of tinnitus retaining and sound therapy, or Cognitive Behavioral Therapy (CBT). One positive aspect about these therapies is that they can be administered face-to-face as well as delivered via internet or smartphone. Smartphones are especially helpful as they are highly personalized devices, and offer a well-established ecosystem of apps, accessible via respective marketplaces of differing mobile platforms. Note that current therapeutic treatments such as CBT have shown to be effective in suppressing the tinnitus symptoms when administered face-to-face, their effectiveness when being delivered using smartphones is not known so far. A quick search on the prominent market places of popular mobile platforms (Android and iOS) yielded roughly 250 smartphone apps offering tinnitus-related therapies and tinnitus management. As this number is expected to steadily increase due to high interest in smartphone app development, a contemporary review of such apps is crucial. In this paper, we aim to review scientific studies validating the smartphone apps, particularly to test their effectiveness in tinnitus management and treatment. We use the PRISMA guidelines for identification of studies on major scientific literature sources and delineate the outcomes of identified studies.

## 1. Introduction

Tinnitus is a disorder or condition mainly associated with the perception of a continuous ringing sound or noise in the ears. Importantly, the phantom auditory sensation exists in absence of any external sound source. Multiple causes for tinnitus have been identified with hearing loss being one of the most important risk factors for tinnitus. Tinnitus affects approximately 15% of the world’s population. Note that for 2% of its sufferers tinnitus can be enfeebling [1]. Presently, tinnitus is regarded as a condition that involves changes at different levels of the auditory pathway and the auditory nervous system. Changes in tinnitus may also be influenced by psycho-social stress (for example, negative thoughts, work or home related stress, etc.) affecting the emotional status and the auditory system [2,3].

Furthermore, patients who perceive tinnitus also report variations in tinnitus loudness and tinnitus-related distress, as well as individual perception of tinnitus [4]. Some influencing factors of this variability are known [4,5,6], however, most of the causative factors for this variability within the tinnitus population are still unknown. For instance, the tinnitus perception variability can be attributed to changes in the atmospheric surroundings [7] and environmental conditions of the patient [8]. Smartphone-based Ecological Momentary Assessments methods can be helpful in better understanding the tinnitus variability in larger tinnitus populations [9].

Given the current understanding of tinnitus, researchers within the tinnitus community are also investigating smartphone-based solutions for mobile diagnosis, event detection, treatment, and monitoring of patients. Recently, smartphone-based solutions such as smartphone apps have gained significant recognition due to popularity of smart sensors such as mobile brain-imaging techniques, and auxiliary health devices like heart meters and smart wristbands within healthcare [10,11]. Furthermore, smartphone application marketplaces provide an ecosystem that can be easily extended with new apps. The antecedent interest of the research community in closely-related health complications of tinnitus like stress [12,13,14,15], Meniere’s disease [8,16], hearing loss [17,18,19], vertigo [20,21,22], or dementia [23,24] affirms an imminent inclusion of smartphone apps for tinnitus too.

Note that further research to determine the effectiveness of smartphone apps in different domains of healthcare is indispensable [25,26], as there is an indication that the smartphone apps can help patients in maintaining and mitigating their health problems [27,28,29]. Similarly, for tinnitus there exists a plethora of smartphone apps to help patients in mitigating and managing their tinnitus symptoms [30]. Although there has not been a cure or treatment for tinnitus, the usual course of action for treatment of tinnitus involves use of tinnitus retaining, sound, or cognitive behavioral therapies [1]. Although the current mode of tinnitus treatment involves face-to-face administration of the aforementioned therapies, however, they can also be administered using smartphones.

Consequently, in recent years, there has been a notable interest in development of smartphone apps aiming at helping patients for management and treatment of their tinnitus [30]. Few attempts on objective quality assessment of tinnitus-related smartphones apps have been reported [30], the clinical effectiveness of these smartphone apps within the context of tinnitus is still questionable. Although existing literature poses many articles reviewing healthcare apps in a more general context [31], very limited literature exists with a specific focus on tinnitus. While Internet or smartphone based CBT has been critically reviewed [32,33], and its effectiveness has been well-documented and established [34], research on reviewing the effectiveness of smartphone apps offering other therapies such as tinnitus therapy, tinnitus retaining therapy, or sound therapy is still scarce. In terms of tinnitus-related therapies to control tinnitus symptoms, Piskosz [35] highlights the use of sound-related therapy. Hesse [36] aims to assess and review smartphone-app–supported therapies for tinnitus and Deshpande and Shimunova [37] presents an evaluation by highlighting the features of smartphone apps. With the fast-growing development and new apps being developed and published in app market-places, an up-to-date review can contribute significantly to the body of knowledge.

Hence, in this article, we provide a detailed review of studies that have evaluated tinnitus apps, specifically in terms of their effectiveness for tinnitus management and treatment. In particular, we take advantage of the PRISMA guidelines [38] for identification of scientific studies. To do so, we have structured the article as follows: the overall review process is highlighted in Section 2, while the identified results are reported in Section 3 and discussed in Section 4. Before concluding the article, the limitations and potential future directions of proposed review are reported in Section 5.

## 2. Methods

**Figure 1:**
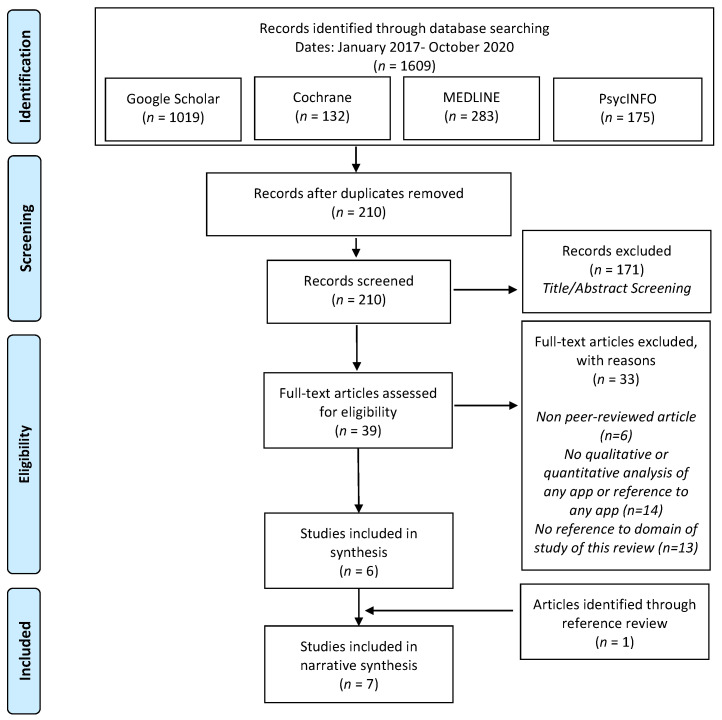
Prisma Workflow for Review.

Several relevant sources for scientific literature were queried for identification of relevant literature based on the PRISMA guidelines. The overall process of identifying, screening, testing for eligibility, and final inclusion of relevant literature is illustrated in Figure 1. The sources were queried with the criteria of finding relevant literature from 2017 onward. The searches were conducted in two separate cycles at different dates—(1) 15 May 2019, and (2) 15 January 2020, and the results were fused together for further screening.

Following criteria were adopted for literature identification:

**Sources:** Primarily, specialized literature database sources like the Cochrane Central Register of Controlled Trials database, MEDLINE, and PsycINFO were searched. Secondarily, Google Scholar was also searched for identification of relevant sources. Since, in recent years, almost all the articles are indexed in Google Scholar, and since it provides wider coverage and better recall, Google Scholar was also considered in addition to specialized electronic database sources.
**Keywords:***tinnitus AND (smartphone OR mobile) AND (Apps OR applications)*
**Search Duration:** To ensure inclusion of most recent articles, the search was limited between the dates of January 2017 and October 2020.
**Inclusion/Exclusion Strategy:** Primarily, manual screening and investigation of manuscript title and abstract, secondarily, full-text assessment.
**Inclusion criteria:** (1) manuscripts with English language, (2) published in peer-reviewed scientific journal, (3) manuscript clearly addressing the subject matters of tinnitus, CBT, or self-help.
**Exclusion criteria:** (1) non-peer reviewed articles, (2) no qualitative or quantitative analysis of any tinnitus smartphone app, (3) manuscripts reporting on technical details about development of the app, but with missing clinical evaluation.
**Intervention:** Studies concerning smartphone-delivered sound therapy, or CBT for treatment and management of tinnitus were eligible. Therefore, the interventions were smartphone or mobile health apps targetting tinnitus or accompanying conditions.
**Population:** Patients suffering from tinnitus along-with healthcare professionals are the primary population. Secondarily, people suffering from stress, anxiety, and depression (commonly occurring comorbidities with tinnitus), as well as the healthcare professionals were also considered.
**Outcomes:** The main outcome of interest for this review is improvement and relief from tinnitus symptoms and accompanying conditions. Therefore, effectiveness of smartphone apps for treatment and management of tinnitus was the target outcome.
**Study Designs:** Mixed method studies aimed to evaluate effectiveness of smartphone apps for tinnitus treatment and management were acceptable and eligible study designs. Pilot studies were also included.

From Figure 1, after removal of duplicates, n=210 records were identified in the identification phase. Next, the titles and abstracts screening of these 210 selected records for eligibility resulted in the feasibility of n=39 records for further evaluation. The full-texts of the selected 39 records were then assessed for further suitability, resulting in a rejection of additional 32 records, due to several reasons: 5 out of the 32 records were not subjected to a peer-review process, 14 records did not perform any qualitative or quantitative analysis of the respective app, or did not reference any app and 13 records did not show any meaningful overlap with the content, aim and scope of this review. The review selection process yielded a total of n=6 records, whereas 1 additional article was added through a manual review of references, the total number of included records was therefore n=7.

In an additional step, we opted to search for relevant literature in aforementioned literature databases using app names. For this process, primarily, we performed an open keyword search on two of the most prominent app markets, namely Google’s Play Store and Apple’s App Store to cover both major mobile platforms (i.e., Android and iOS). We used the keywords tinnitus, hearing, noise, CBT, self-help to search the apps. After carefully screening of app titles as well as app description available on the respective app stores, the search yielded a total of 36 valid tinnitus apps. Secondarily, we performed searches on three independent third-party mHealth app libraries that are: (1) government funded National Health Service (NHS) Apps Library (https://www.nhs.uk/apps-library/ Accessed: 15 January 2020), (2) privately funded AppScript (https://www.appscript.net/ Accessed: 15 January 2020), and (3) privately funded MyHealthApps MyHealthApps (http://myhealthapps.net/ Accessed: 15 January 2020). These third-party mHealth app libraries are inherently web-portals targeted towards enlisting curated smartphone apps [39]. This ancillary search of smartphone apps on third-party mHealth app libraries resulted in identification of additional 6 new apps. Finally, a total of 37 valid tinnitus apps were identified. The names of the identified apps were then used to cross-search any additional study on all previously mentioned scientific literature sources. The overall process did not yield any new literature study, and therefore, the total number of records reviewed in this article remain n=7.

### Quality Assessment

The overall quality of each individual included study was assessed using the *RoB 2: A revised Cochrane risk-of-bias tool for randomized trials* [40]. The Cochrane risk of bias assessment is a valid method for quality assessment for the randomized trials [41]. The tool allows assessment of overall bias as well as biases across following 5 domains:

**D1:**  Bias arising from the randomization process
**D2:**  Bias due to deviations from intended interventions
**D3:**  Bias due to missing outcome data
**D4:**  Bias in measurement of the outcome
**D5:**  Bias in selection of the reported result

Each aforementioned risk of bias domain was classified as ‘High’, ‘Some concerns’, ‘Low’, and ‘No Information’.

## 3. Results

The list of commercially available apps for tinnitus in Google’s Play Store and Apple’s App Store, searched through respective app market places and independent third-party mHealth app libraries are given in Table 1 (Sound Therapy and Tinnitus Management) and Table 2 (CBT). Both Table 1 and Table 2 provide the app name, a small description of the app, as well as the availability on the two major platforms (i.e., Android or iOS). An asterisk (*) in front of the app name corresponds to the app name being reported in the literature without any clinical validation, for instance, technical description about the app development, while apps that are evidence based, reported in literature with clinical evaluation, and reviewed in this article are marked with a dagger (†).

**Figure 2:**
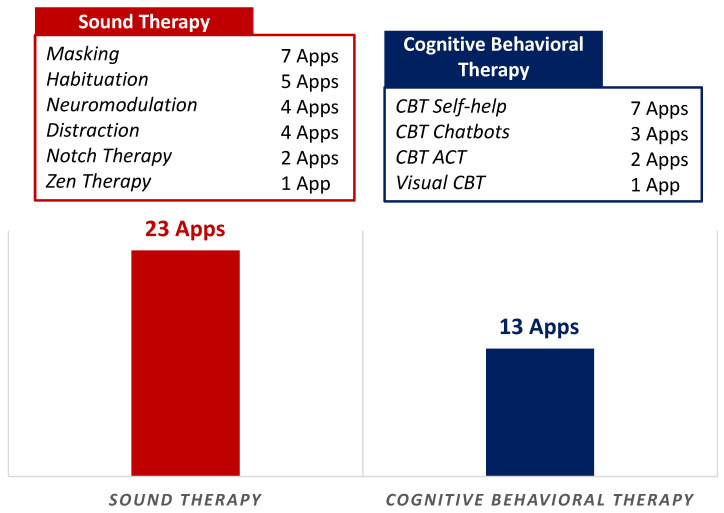
App Types and Categories.

An arbitrary categorical distribution of the 36 identified commercially available smartphone apps with 2 major categories and types is depicted in Figure 2. Based on the app title and description in the app store, we identified 2 main categories: (1) Sound Therapy (23 apps), and (2) CBT (13 apps). In the ‘Sound Therapy’ main category, 7 apps offered tinnitus masking, while 5 apps offered tinnitus habituation (habituation is the process of gradually enabling tinnitus sufferers’ to find ways to emotionally and psychologically cope with tinnitus sounds to find relief). Similarly, 4 apps provided acoustic neuromodulation, 4 apps offered distraction from tinnitus using customized sounds, 2 apps delivered notched therapy for tinnitus relief, and 1 app used zen sounds to offer relief from tinnitus. ‘CBT’ apps for tinnitus made up the second main category, in which 7 apps provided CBT self-help, 3 apps were CBT chatbots, 2 apps offered CBT-Acceptance and Commitment Therapy (ACT), and 1 app used Visual CBT for tinnitus relief.

The initial screening of 210 articles and full-text assessment of 39 articles yielded a total of 7 articles that fulfilled the review criteria and were included in the analysis. All 7 scientific studies are profiled and delineated in Table 3, along-with the characteristics of the study, and the strategy adopted to validate the smartphone app. Furthermore, Table 3 reports on the final outcomes and results of the study. All 7 studies reviewed in this article reported the qualitative analysis using questionnaires like the Tinnitus Handicap Inventory (THI) [42], Tinnitus Functional Index (TFI) [43], Patient Health Questionnaire (PHQ) [44], or ratings scales such as Generalized Anxiety Disorder Scale (GAD-7) [45]. The quantitative analyses results are reported using Latent Dirichlet Allocation (LDA) model [46], or Statistical Package for the Social Sciences (SPSS) program [47]. None of the scientific studies included in this review reported on any side or adverse effect related to smartphone-delivered treatments.

In terms of sound-related therapies for tinnitus management and treatment, Table 3 presents three studies [48,49,50] on the effectiveness of three apps (Tinnitus Therapy Lite, ReSound Relief, Audio Notch). Among these, we can note that there is only one study with appropriate number of patients to be considered to provide significant results [50]. The study presented in Kim et at. [50] employs the use of a smartphone app to deliver tailor-made notched music to tinnitus patients, resulting in improvement of overall THI scores (emotional score of THI in particular) of tinnitus patients. From Kim et al. [50], as Ginkgo Biloba was administered complimentary in combination with tailor-made notched music therapy, it is hard to determine the stand-alone effectiveness of either of the treatment. Regardless of having lower number of patients, the studies presented in Sabarish and Kruthika [48] and, Tyler et al. [49] do report on reducing the tinnitus-related distress after receiving sound therapy. Tyler et al. [49] aims at understanding the effects of delivering sound therapy using the Resound Relief (updated to Resound Tinnitus Relief) app to cochlear implant patients. The results show that 3/10 home trial participants rated the app and sound therapy in reducing tinnitus with high (70%) effectiveness, another 3/10 participants reported the app with moderate (20–40%) effectiveness, another 3/10 participant rated the app with low (0–20%) effectiveness, while 1/10 patients found the app to be completely ineffective.

Furthermore, Table 3 presents three studies [51,52,53] on the effectiveness of three different CBT apps (Wysa, Woebot, MindShift). In terms of depression, the Wysa app showed promising results in reducing depression in patients with severe symptoms with continuous usage of the app. Reduction in PHQ (PHQ-9 and PHQ-2) scores were reported by the patients after usage of the Wysa app [51]. The Woebot app showed significant reduction of depression scores in comparison to patients using the CBT book [52]. In terms of anxiety, the MindShift app reduced anxiety in students after 3 weeks of usage, where the app was found to be satisfactory in terms of usability and acceptability [53].

Finally, Henry et al. [54] reports on the effectiveness of the Tinnitus Coach smartphone app offering the coping skills education program of the Progressive Tinnitus Management (PTM). Henry et al. [54] reports that 8/25 study participants had reduced TFI scores, an indication that the app was beneficial in moderately reducing tinnitus symptoms. Although the participants found most of the content of the app favorable, some features of the app were found to be too complex. Nevertheless, most of the app users suggested that their quality of life improved due to the coping skills taught as part of PTM program. Herein, please note that the Tinnitus Coach smartphone app is not commercially available on either Google’s Play Store or Apple’s App Store.

**Figure 3:**
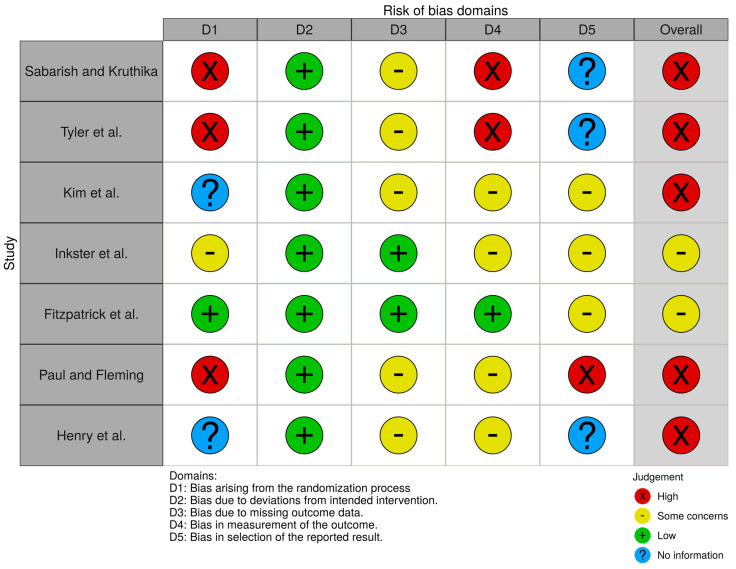
Risk of Bias Assessment—Traffic Light Plot [55].

**Figure 4:**
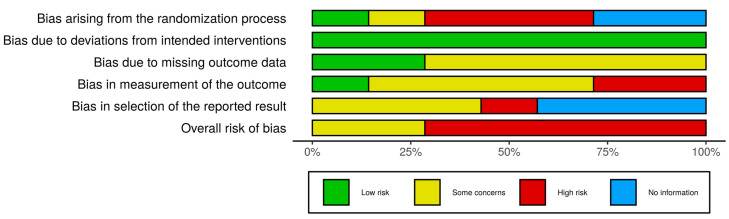
Risk of Bias Assessment—Summary Plot [55].

The Figure 3 shows the results of risk of bias assessment for individual studies with the help of a traffic light plot, while the summary plot for risk of bias assessment results is depicted in Figure 4. Both plots were generated using the robvis tool [55]. Based on the outcomes of the over-all assessment (Figure 3), only 2 out of 7 studies [51,52] had ‘Some concerns’ of bias, while the rest of the 5 studies had ‘High risk’ of bias. Please note that since it is almost impossible to conceal the type of intervention from patients and healthcare providers in case of mhealth interventions, the domain ‘D2: Biases due to deviations from intended interventions’ was assessed at ‘Low risk’ for all identified studies (Figure 4).

## 4. Discussions

The aim of this study was to review scientific studies reporting on effectiveness of smartphone apps used for the management and treatment of tinnitus. The literature identification process resulted in inclusion of 7 scientific studies, for which Table 3 shows the outcome of the included studies. Albeit smartphone apps for tinnitus have been around and available on different platforms for a long time, the amount of research to validate the apps as well as to evaluate the clinical effectiveness of the apps is limited. In our analysis of the identified scientific literature, only three clinical scientific studies pertaining to tinnitus-related therapies, four scientific studies related to CBT therapies were found. From our review of existing literature, we determined that most of the studies with regards to smartphone apps either report on the development, design, implementation, or adoption of smartphone apps in the context of tinnitus. We believe that there is still need of research to be done to clinically evaluate and validate the effectiveness of smartphone apps for tinnitus treatment and management.

Smartphone technology offers a large variety of functions that can be used for the clinical interventions and diagnosis in the chronic tinnitus. For instance, smartphone apps can be used to provide structured counseling to the patients via textual, auditory stimulation using notched music interventions [56] or video information and practical tips. Tailor-made notched music therapy has proven to be an effective treatment modality in reducing tinnitus-related loudness and auditory cortex activity, specifically, while administered for a longer time duration [56,57]. For the diagnosis of tinnitus, Ecological Momentary Assessment (EMA) approaches have been used by several groups to analyze the dynamic changes of the tinnitus symptoms [58].

Different treatment modalities for management of tinnitus symptoms exist, for instance, tinnitus retaining and sound therapy, tinnitus masking, conventional drug delivery, and even brain stimulation—among them, tinnitus retaining therapy, tinnitus masking, or sound therapy using sound generators and CBT as counseling, are standard treatment procedures [1]. Most of the tinnitus relief apps that are generally published on app markets offer tinnitus masking, or sound therapies using different sound techniques like acoustic neuromodulation, notched sound, or amplitude modulation. Importantly, it is significant to note that the smartphones are capable of delivering acoustic and sound therapy reliably and accurately [59].

In addition to sound and tinnitus therapies, CBT has been pivotal for the treatment of tinnitus [60]. It is argued that CBT has no effect on the acoustic characteristics of tinnitus, such as subjective loudness of tinnitus [61,62]. CBT has proven to be effective in improving the overall quality of life of tinnitus patients and reducing symptoms of tinnitus-related psychological comorbidities, such as depression and anxiety [61,63]. Besides CBT being administered face to face with a CBT clinician, it can also be administered via the internet or smartphone as self-help treatment for tinnitus [64]. Evidence from the literature suggests that internet-delivered self-help tinnitus treatment shows positive results and it is an effective treatment modality [65,66]. Consequently, the smartphone app markets have a variety of apps that are specifically designed for CBT for tinnitus, such as Beltone Tinnitus Calmer, Diapason for Tinnitus, ReSound Relief.

Besides, sound therapy and CBT, Progressive Tinnitus Management (PTM) program has recently gained momentum as a possible alternative method for tinnitus treatment and management [67]. PTM is a multi-leveled interdisciplinary care program involving audiologists and mental health providers to offer tinnitus management [67,68]. Level-3 of PTM program serves as an educational program to teach tinnitus sufferers different coping skills for self-management of tinnitus-related distress [69]. It also includes education about different sound therapies taught by audiologists, and delivery of CBT by mental health providers. A randomized control trial showed that coping skills taught as part of the PTM are effective in reducing tinnitus-related distress [70]. The coping skills education program of the PTM can be carried out remotely using videoconferencing [67] and smartphones [54].

Based on the findings of this review and in our opinion, all three studies [48,49,50] providing sound-related therapies for tinnitus management and treatment (Table 3) have predominantly positive findings in relation to tinnitus treatment and management, an indication that smartphone-delivered sound therapy apps can have positive impact on reducing tinnitus-related distress in patients suffering from tinnitus. However, it is also notable that only 3 out of 23 identified commercially available apps (Table 1) providing sound therapy for tinnitus relief have been so far validated, thus prompting the need for further research.

Similarly, three studies [51,52,53] validating the effectiveness of CBT apps (Table 3) reported no direct results in relation to tinnitus treatment or management, instead, the primary focus was on anxiety and depression. However, the limited number of found studies evaluating CBT apps have a notable number of positive results, thus indicating that app-based CBT interventions can definitely help patients to cope with their depression and anxiety problems. Despite the fact that the effectiveness of CBT is well-documented and established for several anxiety disorders [34], additional research is further recommended to understand the effectiveness of CBT in tinnitus related depression and anxiety [61]. Although, the current evidence suggests that internet or smartphone delivered CBT treatment for tinnitus is an effective modality [60,66], from the presented literature review, we establish that studies related to validating the effectiveness of smartphone-delivered or app-based CBT treatment, specifically for tinnitus, are critically not well represented.

Overall, the knowledge about the effectiveness of mobile and smartphone apps in mitigating tinnitus symptoms is very limited and is at its early stage. On one hand, our review process identified that there is a plethora of smartphone apps for tinnitus treatment and management, and obviously the interest to develop and publish new apps will certainly increase, however, the research to clinically validate the smartphone apps is very limited. On other hand, from the limited number of studies included in review, it can be safely concluded that the current clinical role of smartphone apps for tinnitus treatment and management appears promising. Care should be practiced in interpreting the findings of this review, further, the following limitations should be considered. There was a significant risk of bias for all included studies across all domains. There were very limited studies, among which only 4 out of 7 specifically targeted the tinnitus outcome. Finally, the studies included in studies ranged from observational studies, pilot studies, mixed-method studies, all the way to RCTs, although, this allows an exhaustive inclusion of contemporary findings, at the same time it can also influence the over-all synthesis of the results of the review.

## 5. Conclusions

The review presented in this paper thoroughly attempted to highlight the impact of smartphone and mobile health applications, specifically within the context of tinnitus research. Our review approach used the PRISMA guidelines to identify and select the relevant scientific studies. In order to ensure inclusion of relevant literature, we performed searches on market places of prominent mobile platforms (iOS and Android) and the three independent third-party mHealth app libraries to find commercially available smartphone apps for tinnitus. The app names were thus used to find additional literature. Overall, 7 scientific studies validating 7 smartphone apps were identified and reviewed. Based on this, through these measures we were able to (1) comprehensively capture the wide array of heterogeneous apps utilized in tinnitus management and treatment and (2) review and highlight the clinical effectiveness of smartphone-delivered tinnitus management and treatment.

**Limitations**—We understand the limited coverage of keyword-based search as there might be additional relevant documents not matching the chosen keywords. We addressed this issue by isolating keywords that caused reduced recall, however, we still believe that it can be further improved. Furthermore, we thoroughly ensured the selection of relevant literature based on primarily investigating the abstract and introduction for relevance, and secondarily based on the content of the paper. Again, we understand that this approach is subjective and highly relies on the knowledge of the inspector about the domain and can be further improved by collecting opinions from additional domain experts. During our searches, we identified apps, which were relevant for this review and were part of Google’s PlayStore or Apple’s AppStore at one point in time, however, they were removed from respective app stores due to policy conformation issues. Usually, removal of an app from these app stores is properly justified, however, these restrictions can sometimes be inconsistent.

**For future work,** we primarily aim to extend our work by reviewing internet- and computer-based behavioural therapies applied directly in the context of tinnitus research. Herein, an additional focus would be to include studies that report on use of auxiliary and peripheral sensors in assisting therapeutical solutions. For instance, the use of smartwatches or wristbands to acquire physiological attributes of patients suffering from tinnitus could be additionally included. Furthermore, we aim to employ app evaluation and assessment instruments like Mobile Application Ratings Scale (MARS) [71] and the THESIS app evaluation instrument [72] to study the objective quality of the smartphone apps.

## Figures and Tables

**Table 1:** Apps providing tinnitus-related relief using sound therapy (Retrieved: 15 January 2020).

App Name	Description	Platform
Audio Notch *†	Allows creation & listening to customized Notched Sound Therapy	Android, iOS
H & T Sound Therapy	Noise Player (pink noise, white noise or brown noise) for masking tinnitus	Android
Kalmeda mynoise *	Offers medically-based, individual tinnitus therapy	Android, iOS
myNoise *	Controlling tinnitus via combination of different sounds and noises	Android, iOS
Relax Noise 3 *	Masking tinnitus by using red, white, or pink noise	Android
SimplyNoise *	Controlling and managing stress and tinnitus using white, and brown noises	Android, iOS
Starkey Relax *	Tinnitus masking, self-management, and education app	Android, iOS
StopTinnitus *	Masking tinnitus using customised tones	Android, iOS
Tinnitus Aid	Management of tinnitus symptoms by sound therapy specific to tinnitus frequencies	iOS
Tinnitracks *	Controlling and managing tinnitus by filtering out music for sound therapy	Android, iOS
Tinnitus Balance App *	Controlling tinnitus using customized sounds or music	Android, iOS
Tinnitus Help *	Tinnitus masking using natural sounds or music	Android, iOS
Tinnitus Notch	Provided custom tailored notch therapy for tinnitus relief	Android
Tinnitus Peace	Offers melodies to match the frequency of tinnitus to reduce its effects	Android
TinnitusPlay	Tinnitus masking using different sound techniques	iOS
Tinnitus Relief *	Controlling tinnitus using information on different relaxation exercises	Android
Tinnitus Sound Therapy	Sound/Acoustic therapy for masking tinnitus	Android
Tinnitus Tailor *	Personalized sound therapy with sounds created using AI.	Android, iOS
Tinnitus Therapy (Lite) *†	Avoiding tinnitus with sound masking and therapy	Android, iOS
Tonal Tinnitus Therapy *	Helps to mitigate symptoms of tonal tinnitus based on acoustic neuromodulation	Android
Whist *	Controlling tinnitus using sounds with adjusted volume, pitch etc.	Android, iOS
White Noise (Lite) *	Masking tinnitus using environmental sounds	Android, iOS
Widex Zen *	Avoiding tinnitus using relaxing zen sounds, and exercises to manage tinnitus	Android, iOS

**Table 2:** Apps providing tinnitus-related relief using Cognitive Behavioral Therapy (CBT) (Retrieved: 15 January 2020).

App Name	Description	Platform
Beltone Tinnitus Calmer *	Combination of relaxation exercise and sound therapy to avoid tinnitus	Android, iOS
CBT Companion	Employs visual tools to learn & practice CBT techniques	Android, iOS
Diapason for tinnitus *	Game-based digital therapy app for tinnitus relief	Android, iOS
MindShift CBT *†	CBT tools to manage and control anxiety	Android, iOS
Moodfit-Stress & Anxiety	Stress & Anxiety management and tracking, offers CBT exercises for relief	Android
Quirk CBT	Self-help CBT app based on ‘three column technique’	Android, iOS
ReSound Relief *†	Avoiding tinnitus using combination of sound therapy and relaxation exercise	Android, iOS
Sanvello-Stress & Anxiety	Audio & Video CBT exercises for anxiety management	Android, iOS
Stress & Anxiety Companion	CBT based visual exercises to manage stress and anxiety	Android, iOS
What’s Up? A Mental Health App	Offers CBT & ACT methods to manage stress, anxiety as well as depression	Android, iOS
Woebot *†	A chatbot for guided CBT to manage stress and anxiety	Android, iOS
Wysa *†	A chatbot offering CBT and DBT techniques	Android, iOS
Youper: Emotional Health *	A chatbot based on CBT and ACT techniques, monitoring and tracking mood changes	Android, iOS

**Table 3:** Identified scientific studies delineated.

Study			
App/Period	Characteristics	Strategy	**Outcome & Results**
Sabarish and Kruthika [48]			
Tinnitus Therapy Lite 45 min usage for 1 month	Clinical evaluation report Convenience sampling 5 patients: 25–35 yo. 3 phases study: *Phase I:* Audiological evaluations. *Phase II:* Tinnitus evaluation. *Phase III:* THI and app evaluations.	Check app effectiveness by checking THI score changes before & after app usage. Patients trained to use app. Clinical evaluation & regular follow-ups.	Outcome: Tinnitus THI scale improved by 1 grade or 18 points. Tinnitus severity dropped by 25–30%. Users reported satisfaction in app usage. App provided tinnitus pitch and loudness understanding. Low number of participants make the results statistically insignificant.
Tyler et al. [49]			
ReSound Relief 2 weeks	Clinical evaluation report Convenience sampling 16 Participants: 36–85 yo. 3 non-tinnitus participants. Tested with Apple’s 6th Gen. iPod, streaming to Cochlear Implant (CI) device. Laboratory Trial: 13 participants. Home Trial: 10 Participants.	Validate the app sounds and pre-trial & post-trial tinnitus on a scale of 0-100 for Cochlear implant users. Laboratory Trial: 5 mins Home Trial: 2-weeks Home Trial participants reported tinnitus loudness and overall effectiveness of sound therapy using a scale from 0–100 via online questionnaires.	Outcome: Tinnitus Laboratory trial participants reported sounds of rain, music & waves as acceptable. Home trial participants reported sounds of insects & pink noise as acceptable. Both groups reported lower post-trial tinnitus. 3/10 home trial participants found the app more than or equal to 70% effective.
Kim et al. [50]			
Audio Notch 13 March–15 March 30–60 min app usage. Ginkgo Biloba treatment: 3 months	Pilot study Random sampling 26 patients, THI >= 18. Ages: 20–65 yo. Specific inclusion criteria. Participants were instructed on how to use the app.	Check for THI improvements. Distress, Depressive mood, & Audiograms were measured prior to the study.	Outcome: Tinnitus Emotional score of THI improved by 11 points. App reported to be effective if patient had higher initial THI. Listening to familiar music gives emotional comfort and eases distress
Inkster et al. [51]			
Wysa 17 July–17 September	Mixed methods study Random sampling 129 patients. Only patients with Patient Health Questionnaire (PHQ) depression score > 6.	Test for app effectiveness focusing on psychological and mental wellness. Compare score difference for high and low usage groups between Pre-PHQ-9 & Post-PHQ-9. Engagement effectiveness using thematic analysis. Mann-Whitney U test for usage effectiveness impact between usage groups.	Outcome: Depression App gives personalised feedback with good experience and is reported as a bit of hard coping with it. Both groups had reduction in PHQ-9 scores. App classified objections with a recall of 62.1%. Users with high usage had better improvements. PHQ-2 also lowered proving that the app is effective for patients with severe symptoms of depression.
Fitzpatrick et al. [52]			
Woebot 17 January–17 February: 2-Weeks	Randomized Controlled Trial Random sampling 70 patients. Avg. age: 22.20 yo. 34 college students using the app. 36 reading only the CBT related book.	Tested as an alternative CBT delivery method. Test for prediction of depression severity using PHQ-9, (Generalized Anxiety Disorder) GAD-7, & Positive and Negative Affect Scale.	Outcome: Anxiety & depression Depression scores of users using the app decreased significantly in comparison to book users. Both groups had lower GAD-7 scores.
Paul and Fleming [53]			
MindShift 3 weeks 5 days/week Minimum 15 min per day usage	Exploratory Study Stratified sampling 104 students: Avg. age 19.83 yo. Selection based on high levels of anxiety seen in PHQ scores.	Test for reductions in anxiety, depression from baseline PHQ-15, GAD-7, and PHQ-9 scores and app acceptance. Check for users feedback via 3 questions about usability and acceptability.	Outcome: Anxiety and depression Reduction in anxiety after 3 weeks usage reported. Users reported satisfaction in app usability and acceptability.
Henry et al. [54]			
Tinnitus Coach 6–8 weeks	Observational field study Stratified sampling 25 participants Every participant received phones with pre-loaded app. Monetary incentives offered.	Test app in a 3-phase study: *1. Design & Develop*, *2. Initial Test*, *3. Evaluate*. Measure Tinnitus Questionnaire and the Tinnitus Functional Index (TFI) after app usage.	Outcome: Tinnitus Insignificant or minor TFI changes were reported, where only 8 participants reported reduced TFI. Users suggested that coping skills taught as part of Progressive Tinnitus Management improved quality of life with tinnitus.
